# Mapping Nursing Competencies Described for Disaster Response Within the Civil Defense Context: A Scoping Review

**DOI:** 10.3390/nursrep16060206

**Published:** 2026-06-18

**Authors:** Gabriele Caggianelli, Marco Iorfida, Fabio Petrelli, Maurizio Fiorda, Marco Ricci, Samanda Pettinari, Francesca Marfella, Roberto Accettone, Valentina Vanzi, Gennaro Rocco, Francesco Scerbo, Stefano Mancin, Maurizio Zega, Giovanni Cangelosi

**Affiliations:** 1 Department of Healthcare Professions, Azienda Ospedaliera Complesso Ospedaliero San Giovanni Addolorata, Via dell’Amba Aradam, 9, 00184 Rome, Italy; caggianelligabriele@gmail.com (G.C.); iorfida.m@gmail.com (M.I.); 2 JBI Italy Evidence-Based Practice and Health Research Center, Viale degli Ammiragli, 67 sc.B, 00146 Rome, Italy; 3 School of Nursing and Midwifery, Sapienza University of Rome, AO San Camillo-Forlanini Hospital, Cir.ne Gianicolense 87, 00152 Rome, Italy; 4 School of Pharmacy, Experimental Medicine and “Stefania Scuri” Public Health Department, Via Madonna delle Carceri 9, 62032 Camerino, Italy; giovanni01.cangelosi@unicam.it; 5 Italian Coordination of Volunteer Nurses for Health Emergencies Association (CIVES), Via Agostino Depretis 70, 00184 Roma, Italy; 6 Azienda Regionale Emergenza Sanitaria ARES 118, Via Portuense, 00149 Rome, Italy; raccettone@ares118.it; 7 Center of Excellence for Nursing Culture and Research, Order of Nursing Professions of Rome, Viale degli Ammiragli, 67 sc.B, 00146 Rome, Italy; genna.rocco@gmail.com (G.R.); maurizio.zega@opi.roma.it (M.Z.); 8 International Center for Nursing Research Montianum Our Lady of Good Counsel Catholic University Tirana, Rr. Dritan Hoxha, 1001 Tiranë, Albania; 9 Board of Nursing Professions of Rome, Viale degli Ammiragli, 67, 00136 Roma, Italy; 10 IRCCS, Humanitas Research Hospital, Via Manzoni 56, 20089 Rozzano, Italy

**Keywords:** civil defense, disaster nursing, disaster management, nurse’s role, scoping review

## Abstract

**Background/Aims:** The increasing complexity of disasters requires effective integration of nurses into Civil Defense (CD) systems. Despite their crucial role, the competencies needed to operate within these multi-agency frameworks remain fragmented and insufficiently defined. The main aim of the study was to map nursing competencies for disaster response within the CD context, identifying essential skills, contextual variations, and barriers to application. **Methods:** A scoping review was conducted following the JBI methodology and reported according to PRISMA-ScR guidelines. Major databases (PubMed, CINAHL, Scopus, Embase) were searched without time limits, resulting in the inclusion of 27 studies published between 2011 and 2025. **Results:** 12 core competency domains were identified. Clinical care was the most cited competency (70% of studies), followed by communication (63%), leadership (60%), triage (48%), and psychosocial support (48%). The lack of specific training emerged as the primary individual barrier (44%), while the absence of standardized curricula was the leading systemic obstacle (41%). Competency requirements varied significantly based on the hazard type and organizational setting. **Conclusions:** Disaster nursing is emerging as an essential specialized field in response to the increasing frequency of climate-related events and global conflicts. There is an urgent need to move beyond purely clinical training to integrate “organizational literacy” and psychological resilience, harmonizing educational pathways with national CD policies and competency-based disaster preparedness programs.

## 1. Background

Disasters are serious disruptions affecting communities and societies that result from the interaction of hazards, exposure, vulnerability and capacity, leading to human, material, economic and environmental losses [1]. The Sendai Framework for Disaster Risk Reduction 2015–2030 highlights the importance of a shared understanding of disaster risk and coordinated management across sectors and countries [2]. Recent data indicate increasing disaster impacts worldwide, with substantial mortality and economic losses despite a slight decline in disaster frequency [3]. Civil Defense (CD) systems play a central role in disaster management. Originally developed to protect civilian populations during armed conflicts, they have evolved into complex structures addressing both natural and human-made hazards [4,5]. These systems involve multiple actors, including governmental agencies, non-governmental organizations, military forces, healthcare professionals and volunteers, operating across different administrative and legal frameworks [6]. This diversity increases operational complexity and underscores the importance of coordination and the effective integration of healthcare professionals within CD structures. Among these professionals, nurses represent the largest segment of the global health workforce and are essential actors in disaster management systems. Their contribution extends across preparedness, response and recovery activities, supporting both population health needs and the operational objectives of CD organizations. Disaster settings differ markedly from routine clinical environments: resources are often limited, infrastructure may be compromised, and care demands are high, requiring adaptability and rapid decision-making [7,8,9]. The scope of disaster nursing has expanded beyond direct clinical care to include preparedness, planning, mitigation, recovery activities, and to contributing across all phases of the disaster management cycle [10]. This expanded role reflects a growing professional and strategic presence within disaster management systems. To support role standardization, the International Council of Nurses (ICN), in collaboration with the World Health Organization (WHO), developed the ICN Framework of Disaster Nursing Competencies, first published in 2009 and updated in 2019 [11]. The framework defines eight domains of competency across the phases of disaster management and three levels of nursing practice, providing an international benchmark for education, research and policy development [8,10]. Disaster nursing competency is commonly conceptualized as the integration of knowledge, skills and attitudes (KSA) [12]. Core dimensions include clinical, professional, cultural and relational competencies, encompassing not only technical care delivery but also ethical practice, cultural responsiveness, communication and coordination within multidisciplinary teams [13]. These competencies are essential for effective functioning within complex CD systems. Despite the availability of competency frameworks and the expansion of disaster nursing education, evidence suggests that many nurses remain underprepared for disaster response. Reported barriers include limited training opportunities, insufficient exposure to disaster scenarios and gaps between education and practice [14].

### Aims and Research Questions

Given the evolving role of nurses, the complexity of CD systems and the diversity of existing competency frameworks, this scoping review aims to map the nursing competencies described in the literature for disaster response within CD, to inform education and training, support workforce planning and clarify the role of nurses within CD systems. The main review question is:

“Which nursing competencies have been reported in the literature for disaster response within CD?”

This scoping review also aims to address the following specific sub-questions:

- What competencies (KSA) are described as essential for nurses involved in different types of disasters?
- How do required nursing competencies vary across disaster types, geographical areas, and organizational settings?
- What challenges or barriers are reported in acquiring or applying these competencies during disaster response?
- On what basis (policies, guidelines, accreditation frameworks, or empirical evidence) are nursing disaster response competency requirements established?

## 2. Methods

This scoping review follows the JBI methodology for scoping reviews [15] and is reported according to the Preferred Reporting Items for Systematic Reviews and Meta-Analysis extension for scoping reviews (PRISMA-ScR) guidelines [16] (Supplementary File S1). This review was conducted in accordance with an a priori protocol registered in the Open Science Framework (OSF) database (https://osf.io/qduyp, 16 April 2025) and previously published [17].

### 2.1. Search Strategy

In order to locate both published and unpublished studies, a three-step search strategy was utilized in this review. After an initial limited search of MEDLINE (via PubMed) and CINAHL (via EBSCO host), the text words contained in the titles and abstracts and the index terms of relevant articles were used to develop a full search strategy for MEDLINE (via PubMed), Cochrane Database of Systematic Reviews (via Cochrane Library), Embase, Scopus, and CINAHL (via EBSCOhost). Additionally, the reference list of all included papers was hand-searched for additional studies.

### 2.2. Inclusion and Exclusion Criteria

The inclusion criteria of this scoping review were based on the Participants, Concept and Context (PCC) framework [15]. This review considered studies on nurses, including registered nurses, nurse practitioners, advanced practice nurses, disaster nurses or volunteer nurses (Participants) and their competencies, including clinical, professional and cultural competencies and social skills (Concept) for disaster response in natural, human-made and multi-hazard disaster, including terroristic events, armed conflicts and epidemics (Context). This scoping review considered quantitative, qualitative and mixed methods studies. In addition, systematic reviews that meet the inclusion criteria, text and opinion papers were also considered for inclusion, depending on the research question. There were no time or language restrictions. The authors had reserved the right to include studies published in all languages, including Chinese, provided that the title and/or abstract was available in English and suggested the study meets the inclusion criteria; however, it did not occur. All manuscripts that did not meet the established criteria were excluded from the selection.

### 2.3. Source of Evidence Selection

The databases were searched by two researchers independently from 27 July to 7 August 2025. All retrieved papers were exported to the web-based tool Rayyan [18], where duplicates were removed and screening and information extraction were undertaken. Detailed search strategies are given in Supplementary File S2. After removing duplicates, reviewers completed a two-step screening process for all retrieved articles in Rayyan. In the first step, titles and abstracts were blind-screened by two authors against the inclusion or exclusion criteria. Potentially relevant sources were retrieved in full and their citation details imported into the JBI System for the Unified Management, Assessment and Review of Information (JBI SUMARI) [19]. The full text of selected citations was assessed in detail against the inclusion criteria by two reviewers. All the disagreements that arose between the reviewers at each stage of the selection process were resolved through discussion with an additional expert reviewer. The results of the search, the study inclusion process and reasons for exclusion of sources of evidence at full text were recorded and reported in full and presented in a PRISMA ScR flow diagram [16]. For reference management the software Zotero (vers. 7.0.11) was used.

### 2.4. Critical Appraisal

A critical appraisal of the selected studies was performed in order to report the risk of bias. However, no article was removed as critical assessment is not mandatory in scoping reviews [15]. Two reviewers independently and critically appraised the included articles, using the JBI critical appraisal tools embedded in the JBI SUMARI software [20,21,22,23]. The results of the appraisal are reported in Supplementary Table S1.

### 2.5. Data Extraction

Data were extracted from articles included in the scoping review by two independent reviewers using a data extraction tool developed by the reviewers, based on the JBI instrument from the Manual for Evidence Synthesis [15] and refined following piloting with a small number of studies and subsequently applied to all included studies. The data extraction tool was refined throughout the process to ensure all extracted data were accounted for. Any disagreements that arose between the reviewers were resolved through discussion or with an additional reviewer. Data were extracted in a specific preliminary table. No authors were contacted to request additional data.

### 2.6. Data Analysis

After describing the general characteristics of included studies, the extracted data were analyzed and reported using a narrative synthesis to explain how the findings relate to the review objectives and questions. In this review, nursing competencies are conceptualized as integrative constructs encompassing KSA according to Lizzio and Wilson’s study (2004) [24]; accordingly, extracted data were mapped across these domains to reflect how competencies are described and operationalized in the literature. Findings are presented in both narrative and tabular formats to illustrate their alignment with the review objectives.

## 3. Results

### 3.1. General Characteristic of Included Studies

**Figure 1:**
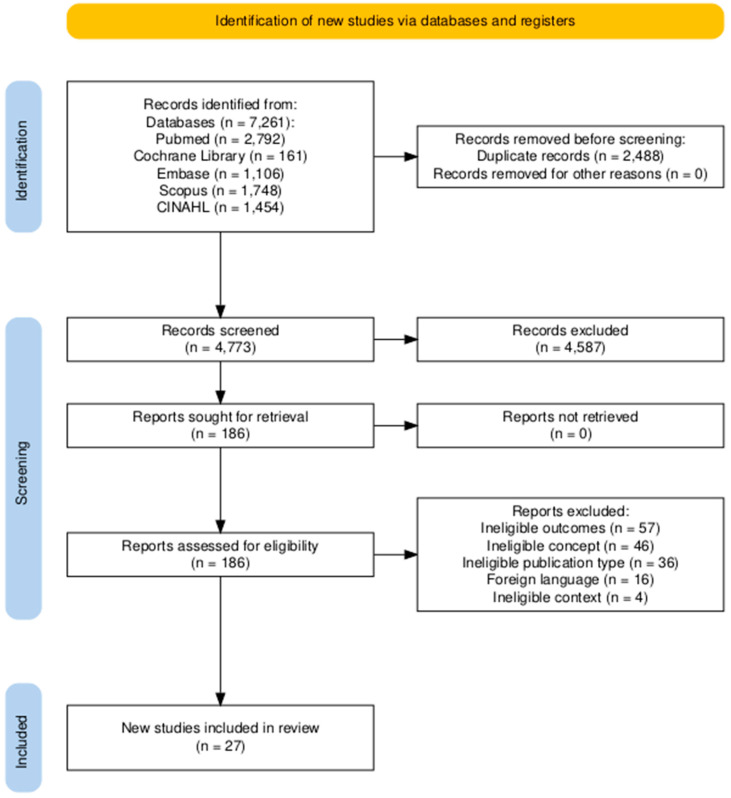
PRISMA flow chart.

The search strategy generated a total of 7261 references. After exclusion of duplicates, 4773 titles were identified for further screening. A total of 4587 records were excluded after title and abstract screening according to the exclusion criteria. This resulted in 186 full-text articles being assessed for eligibility; 159 did not meet the inclusion criteria, leaving 27 references for analysis [25,26,27,28,29,30,31,32,33,34,35,36,37,38,39,40,41,42,43,44,45,46,47,48,49,50,51]. A visual representation of the study selection process is reported in a PRISMA flow diagram (Figure 1).

The final extraction table included 27 references [25,26,27,28,29,30,31,32,33,34,35,36,37,38,39,40,41,42,43,44,45,46,47,48,49,50,51] published between 2011 and 2025, summarized in Table 1. The most represented countries in which the studies were conducted were the United States (six studies) and China (five studies), followed by Brazil and Iran (four studies each); there are also contributions from Saudi Arabia, Turkey, Portugal, Peru, Indonesia, and Australia. One was conducted between China and Japan. The study designs included in the review are varied and include 10 qualitative studies (seven on semi-structured interviews, three focus group papers), five scoping reviews, five expert consensus papers (mainly Delphi method), four opinion studies, two cross-sectional surveys, and one integrative review. The population investigated is predominantly composed of nursing staff (nurses, registered nurses, emergency nurses, primary healthcare nurses, forensic nurses, disaster nurses), with some studies aimed more broadly at healthcare teams [33,49] or public health personnel [39]; there are also studies involving, in addition to nursing professionals, hospital administrators [43] and medical doctors in specific contexts [35,50]. The settings and types of disasters considered varied considerably. Fourteen studies adopted an all-hazards approach without focusing on specific types of disasters. Among the remaining studies, most focused on natural disasters: five studies dealt exclusively with earthquakes, one with floods, one adopted a specific focus on earthquakes but generalized the results to all types of disasters, and one included earthquakes, floods, and pandemics. Three studies focused on conflict or war-zone contexts; one on biological disasters, both natural and anthropogenic; and one on disasters in urban contexts. Twenty-one studies do not report specific organizational settings, five apply to hospital settings, and one applies to both hospital and pre-hospital settings.

### 3.2. Nursing Competencies Described as Essential for Disaster Response

Essential nursing competencies in disaster response encompass a diverse array of competencies mapped within the KSA framework distributed across 12 core domains. As synthesized in the competency matrix (Supplementary Table S2), these domains vary significantly in their reported frequency across the literature. Clinical care is the most frequently identified essential competency, appearing in 70% of the included studies. Twenty-four of the 27 studies include clinical, communication or leadership as core requirements. While communication (63%) is often described as a practical skill for interdisciplinary coordination, leadership (60%) is frequently viewed as a combination of both theoretical knowledge and practical application. Specific clinical skills were notably emphasized in earlier field-based research [51]. Both triage and psychological support are recognized as essential in 48% of the studies. Triage is predominantly characterized by the application of rapid assessment skills, whereas psychological support frequently integrates all three KSA dimensions, with a particular emphasis on the attitude required to manage victim trauma and distress. Ethical practice is reported in 41% of the studies, often appearing as a synthesis of theoretical knowledge and professional attitude. This is particularly evident in studies focusing on high-pressure environments [47] or conflict zones [31]. Competencies related to specific hazards and supportive organizational functions are reported with lower frequency: infection prevention and control and Chemical, Biological, Radiological, Nuclear, and Explosive (CBRNe) hazards are each noted in 26% of the studies; cultural competence and surveillance are described as essential in 22% of the literature; documentation and advanced competencies were reported less frequently than the other domains and were synthesized in the competency matrix together with the remaining competency areas (Supplementary Table S2). Overall, while the specific emphasis varied by context, the literature converged on a core set of clinical, organizational, and personal competencies required for nursing disaster response.

### 3.3. Variability in Nursing Competencies Across Disaster Types, Geographical Areas and Organizational Settings

Disaster nursing competencies are dynamic, shifting significantly according to hazard types, geographical regions, and organizational settings, as shown in Supplementary Tables S3–S5. Specific hazards dictate priority domains: studies on earthquakes emphasize clinical care, triage, and psychological support [28,51]; floods emphasize infection control and surveillance; while armed conflicts require ethics and advanced care [31]. Biosafety incidents prioritize strict safety compliance and documentation [27]. Regionally, Asia integrates cultural and psychological support [36,51], while the Middle East specializes in triage and ballistic trauma management [27,31]. The South American literature highlights leadership and forensic aspects [32,45], whereas Western contexts focus on standardized protocols and Incident Command Systems (ICSs) [39,43]. Work environments further define the KSA mix: hospitals prioritize clinical care, leadership, and surge capacity management [25,42]; pre-hospital/field settings demand high autonomy, field triage, and logistical improvisation [40,46]; community care focuses on long-term psychological management and vulnerable populations [36,41]; military/austere settings require specialized “Prolonged Casualty Care” and advanced skills rarely cited elsewhere [27,32,35].

### 3.4. Challenges and Barriers in Acquiring or Applying Disaster Response Competencies

The acquisition and application of disaster nursing competencies are hindered by a multi-level array of challenges, categorized into individual, organizational, and systemic barriers (Supplementary Table S6). A lack of prior training or specific disaster experience is the most frequent individual barrier, reported in 44% of the literature. Twelve of the 27 studies identify this as a primary obstacle to competency. Additionally, psychological and moral distress represents a significant hurdle, reported by 30% of the studies, particularly in high-intensity contexts such as earthquakes and conflict zones. At the organizational level, resource scarcity and role ambiguity (including a lack of clear protocols) are each identified by 22% of the studies: six studies highlight how a lack of essential supplies limits the practical application of clinical skills, and six studies identify unclear command structures as a barrier, with specific instances noted in earlier Iranian and Brazilian contexts [45,48].

Systemically, inconsistent educational frameworks remain a major obstacle, affecting 41% of the contexts studied. Eleven studies identify the lack of a standardized curriculum as a root cause of knowledge gaps. Finally, cultural and language barriers are cited in 15% of the literature, particularly in humanitarian or diverse regional settings [28,49].

The identified barriers should not be interpreted as isolated challenges. Rather, they appear mutually reinforcing. For example, the absence of standardized curricula may contribute to training deficiencies, which in turn can hinder nurses’ preparedness for multi-agency disaster response. Addressing these barriers therefore requires coordinated interventions at both educational and organizational levels [25,26,27,28,29,30,31,32,33,34,35,36,37,38,39,40,41,42,43,44,45,46,47,48,49,50,51].

### 3.5. Bases for Nursing Disaster Response Competency Requirements

Analysis of the included studies reveals that nursing disaster competency requirements are based on several methodological and theoretical foundations, classified into empirical evidence, international guidelines, accreditation frameworks, national policies, and expert consensus (Supplementary Table S7). Empirical evidence is the most common foundation, used in 14 studies to derive competencies from field experience or literature reviews. Eight of these studies rely exclusively on empirical data—such as qualitative interviews, post-disaster surveys, or systematic reviews—while the remaining six integrate empirical evidence with other frameworks. Expert consensus, typically achieved through the Delphi method or dedicated task forces, was used in three studies to define standards for specific areas like biosafety or all-hazard response [26,43,50]. International guidelines, specifically the ICN Core Competencies in Disaster Nursing, serve as the foundation for eight studies, confirming their status as the “gold standard” for global standardization [30,42]. Professional accreditation frameworks and health policies are also frequently utilized: five studies base their requirements on professional board certification standards or established institutional methodologies; national health strategies and governmental mandates drive competency definitions in five contexts, often combined with international guidelines to align requirements with local health systems [43,45,47]. This multiplicity of bases reflects a literature transition from models based purely on observational experience toward more structured and institutionalized frameworks.

## 4. Discussion

**Figure 2:**
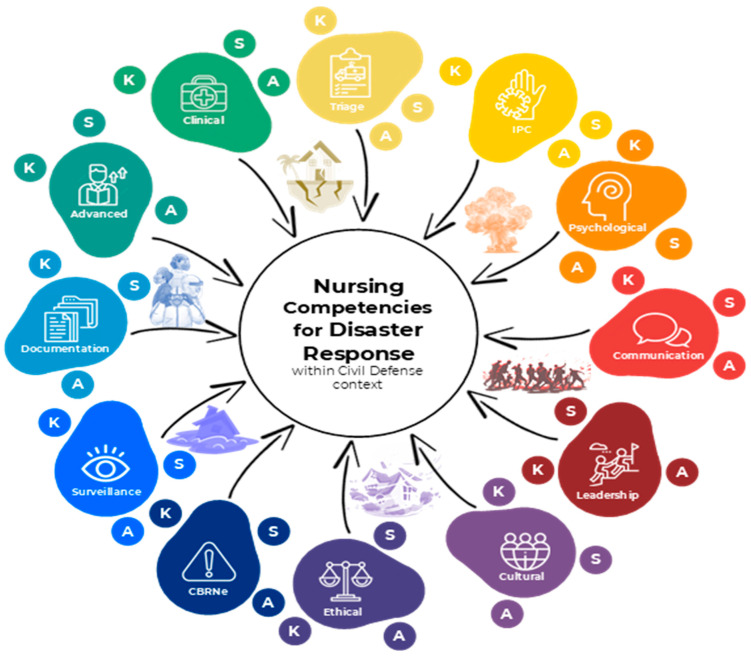
Disaster nursing competences.

This scoping review mapped nursing competencies for disaster response within the CD context, analyzing studies that emphasize the multidimensional nature of disaster nursing (Figure 2). Our findings indicate that these competencies are not merely a collection of technical tasks but a complex integration of KSA across clinical, organizational, and relational domains [24].

A central result of this mapping is the predominance of technical–clinical competencies, particularly those related to triage, first aid, and mass-casualty management. Within the CD context, this emphasis reflects the expectation that nurses operate as immediate responders during the acute phase of disasters. As noted by Firouzkouhi et al. (2021) [52], clinical decision-making during the first 72 h has the greatest impact on reducing morbidity and mortality. Furthermore, the studies included in this review suggest that higher levels of disaster-specific clinical competence are associated with increased professional confidence, a relationship also reported in recent international studies [53,54]. Beyond clinical skills, our results underscore the strategic importance of organizational and coordination competencies. In CD operations, nurses must integrate into hierarchical and often militarized structures, such as the ICS. This requires what can be termed “organizational literacy.” The literature suggests that coordination breakdowns represent an important contributor to challenges in disaster response, alongside technical and clinical limitations [55]. Specifically, studies in the European context highlight that insufficient familiarity with CD procedures can impede nurses’ operational effectiveness, regardless of their clinical expertise [56]. Relational and communication competencies also emerged as critical, particularly when collaborating across civilian and military sectors [57]. Nurses frequently act as mediators between institutions and vulnerable populations, requiring advanced negotiation, communication, and leadership skills [58]. In addition, several studies included in this review emphasized the relevance of psychosocial and cultural competencies. This finding aligns with evidence suggesting that psychological first aid and culturally sensitive care are important components of effective responses during prolonged crises [59]. Qualitative evidence from volunteer nurses further demonstrates how these relational skills can support team cohesion and trust-building in unstable humanitarian conditions [60]. Nevertheless, the relative importance assigned to different competency domains varied across the included studies. While some authors primarily emphasized technical–clinical preparedness, others highlighted organizational, leadership, psychosocial, or communication competencies as equally essential. This variability may reflect differences in disaster typologies, healthcare systems, operational settings, and the specific roles nurses are expected to assume within CD structures [25,26,27,28,29,30,31,32,33,34,35,36,37,38,39,40,41,42,43,44,45,46,47,48,49,50,51].

Despite the identification of these core competencies, our review also reveals persistent barriers to their development. Many nurses, particularly those involved in volunteer organizations, are mobilized without standardized training pathways or sufficient opportunities for experiential learning. This challenge has been documented across different geographical settings and has been associated with role ambiguity, reduced preparedness, and increased psychological stress among responders [61]. Furthermore, although clinical competencies are frequently prioritized in educational programs, psychosocial and organizational competencies appear to be less systematically integrated, despite evidence suggesting their positive contribution to both population outcomes and healthcare workers’ wellbeing [62]. At the same time, important gaps remain in the current literature. In summary, the transition to disaster nursing within CD requires a comprehensive competency framework that integrates clinical expertise with organizational and relational capabilities. This need is increasingly influenced by structural changes in the global risk landscape: climate change is intensifying extreme weather events and cascading public health emergencies, while political instability and armed conflicts continue to generate complex humanitarian crises. Nurses require targeted and anticipatory preparation that includes disaster-specific competencies, organizational literacy, and psychological resilience, while future research should focus on developing more standardized competency frameworks and evaluating their impact on operational outcomes.

### 4.1. Implications for Research, Education and Practice

The implications of this scoping review delineate a necessary roadmap for evolving the nursing role from a generalist practitioner to a specialized asset integrated within CD systems, supported by a reform of educational pathways. In terms of professional practice, it is clear that clinical excellence is no longer sufficient unless accompanied by a robust organizational literacy that allows professionals to navigate effectively within a complex command and control structure [63]. This requires healthcare institutions to move beyond viewing nurses merely as personnel to be deployed during emergencies; instead, they must be actively involved in the planning and mitigation phases, with clearly defined protocols to reduce the role ambiguity that was identified as a primary driver of psychological distress [64]. The educational pathways should move away from an exclusive clinical focus toward a multidisciplinary approach. It is essential that training includes joint simulations with military forces and volunteer organizations, aiming to develop the relational skills and interprofessional leadership [65,66]. Furthermore, education must systematically integrate psychological first aid and cultural sensitivity, preparing staff for the complexities of modern migration and humanitarian crises [67]. In this context, future scientific research is called upon to move beyond the mapping of perceived competencies and focus on longitudinal studies that measure the actual impact of nursing interventions on patient outcomes during disasters [68]. Additionally, it is necessary to investigate the effectiveness of psychological “pre-habilitation” and resilience interventions to protect the wellbeing of responders, given the high emotional burden [69]. Only through synergy between research, education, and practice will it be possible to develop a universal competency framework capable of meeting the challenges of a constantly evolving disaster landscape [70].

### 4.2. Limitations

This scoping review was conducted on the basis of a pre-registered protocol and in accordance with established methodological guidance, contributing to its transparency, traceability, and scientific rigor. Furthermore, the adoption of a broad and structured search strategy proved particularly appropriate for a field characterized by marked conceptual heterogeneity. An additional strength lies in the explicit focus on the CD context, which distinguishes this review from the existing literature that often addresses disaster nursing in more general terms. Despite these strengths, several limitations should be acknowledged. Consistent with the exploratory nature of scoping reviews, this study did not assess the effectiveness or the relative importance of the identified competencies, but rather aimed to map and describe the available evidence. Consequently, the findings do not support the establishment of a universal prioritization or ranking of competencies across heterogeneous CD and disaster response contexts. The body of included studies is characterized by substantial heterogeneity, both in terms of study designs and with regard to the types of disasters examined and the operational definitions of competencies adopted; this limits comparability across findings, complicates any unified interpretative synthesis, and restricts the ability to determine the underlying factors driving observed variations in competency requirements.

Additional concerns arise from the nature of the included sources, many of which rely on self-reported data or expert opinion, thereby exposing the findings to potential biases, including reporting bias and social desirability bias. This is further compounded by a possible selection bias, as most studies originate from high- and middle-income countries (with a substantial proportion conducted in the United States and China); therefore, the generalizability of these findings to low-income settings and healthcare systems with different organizational, technological, and educational infrastructure may be limited. In addition, studies were frequently conducted in hospital or emergency care settings, resulting in the underrepresentation of community-based or resource-limited contexts. Furthermore, the included studies spanned a 14-year publication period, during which clinical decision-making processes, healthcare technologies, and educational priorities have evolved considerably. Consequently, some competency frameworks may not fully reflect current practice requirements, despite their continued relevance in informing the core domains identified in this review. Furthermore, organizational, regulatory, and operational differences may significantly influence the transferability of the identified competencies, making context-specific adaptation necessary when applying these results to particular national or institutional settings. Finally, the competency matrix was developed by mapping reported competencies onto the KSA framework; while this approach enabled a structured synthesis of the literature, it was not designed to explore the underlying reasons for variations in the frequency of competency reporting or the complex interrelationships among competencies. Future research should further investigate these aspects to better understand how competencies interact and evolve across clinical contexts.

## 5. Conclusions

This scoping review highlights that nursing competencies for disaster response within CD systems constitute a complex, multidimensional construct that extends far beyond traditional clinical expertise. Our findings indicate that while technical skills remain foundational, the ability to operate within hierarchical command structures, facilitate interprofessional coordination, and provide culturally sensitive psychosocial support is a defining component of effective disaster nursing. These results directly address our review objectives by highlighting a persistent gap between theoretical frameworks and the operational realities of multi-agency responses. As climate change continues to drive an intensification of extreme weather events and global instability increases the frequency of armed conflicts, the disaster nurse is rapidly emerging as a critical, specialized role in modern society. There is an urgent need to advance from general emergency preparedness toward structured educational pathways specifically aligned with disaster response within CD systems. The next steps should involve the harmonization of international educational standards with national CD policies, promoting competency-based preparedness and ensuring that nurses are not only clinically prepared but also organizationally and psychologically sufficiently resilient to meet the challenges of an increasingly volatile global landscape.

## Figures and Tables

**Table 1:** Data synthesis.

Authors, Year of Publication/Country of Origin	Aims/Purpose of the Study	Study Design/Methods of Data Collection	Population (n)	Type of Disaster/Setting	Competency
Rizek [25], 2025/USA	Foundational skills, competencies, and strategies for emergency nurses to develop expertise in disaster nursing	Opinion study	Emergency nurses	All disasters/not reported	Clinical skills; infection control; health education; decontamination; psychological support
Wu et al. [26], 2025/China	Nursing staff’s competence to respond to biosafety incidents	Expert consensus/Delphi method	Nurses	Biosafety incidents/not reported	Biosafety infection protection; biosafety incident care management
Mani et al. [27], 2024/Saudi Arabia	Perceptions of core competencies necessary to work in hospitals near armed conflict	Qualitative study/semi-structured interviews	Emergency nurses (15)	Armed conflicts/hospital	Leadership; communication; assessment and intervention
Pierre et al. [28], 2024/Brazil	Analysis of care practices, especially nursing, in earthquakes	Scoping review	Nurses	Earthquakes/not reported	Clinical care; psychosocial care; ethical care; care coordination; victim care network organization; teamwork
Salik et al. [29], 2024/Turkey	Experiences of nurses providing care to individuals in earthquake-stricken areas	Qualitative study/semi-structured interviews	Nurses (11)	Earthquakes/not reported	Initiating life-saving and disease/injury preventive interventions; coordinating healthcare teams; performing triage; providing first aid; informing the affected community; offering psychological support
Santos et al. [30], 2024/Portugal	The meaning of a competence framework for Portuguese general nurses and identifying those that are considered crucial for competent preparedness and response in disaster scenarios	Expert consensus/Delphi method	Nurses	All disasters/not reported	Knowledge of response plan; ability to cooperate and commitment to teamwork; maintenance of safety awareness and towards self and others, adapting basic infection control practices to the available resources; continuous surveillance; maintaining ongoing assessment; implementing basic first aid; providing advanced clinical care; implementing infection control surveillance; isolation procedures; outbreak management; providing patient care based on priority needs and available resources; participating in surge capacity activities as assigned; moral and ethical skills
Mani et al. [31], 2023/Saudi Arabia	The importance of competencies for nurses working in areas of armed conflict	Cross-sectional study/survey	Emergency nurses (163)	Armed conflicts/hospital	Triaging patients; management of surge capacity; ethically allocating scarce resources; critical, flexible and creative thinking; security and safety; infection control practices; CBRNe knowledge and decontamination; providing first aid principles; communication and documentation
Silva et al. [32], 2023/Brazil	Sources of technical–scientific information on Forensic Nursing competencies in disaster situations	Scoping review	Forensic nurses	All disasters/not reported	Risk management; direct care; psycho-emotional care; collection and preservation of traces; registration and documentation with photography; body management; maintenance of the chain of custody; epidemiological surveillance
Lin et al. [33], 2022/China	Identification of the cultural competencies required for disaster nursing in China	Scoping review	Healthcare workers	All disasters/not reported	Cultural desire; cultural awareness; cultural knowledge and skills (communication in multiple languages/considering the local space norms; conformity with the local social organization’s way/cooperation with the key person in the local social organization; compliance with the beliefs and customs on health, nursing and medication of survivors/with religious beliefs and customs of the survivors/with beliefs of death and burial customs of the survivors/with daily life and diet customs of the survivors; respecting the local sense of time; considering biological variations; cooperation with local staff/organizations/governments; incorporating cultural consideration in assessment); cultural encounters
Su et al. [34], 2022/China–Japan	The skills nurses required for different types of disasters	Scoping review	Disaster nurses	All disasters/not reported	Casualty triage; observation and monitoring; basic first aid techniques; psychological nursing; communication skills; common injuries and nursing skills; acute respiratory care; marine infusion care; sea-to-land transfer skills; critical care patient management; infectious disease management; infection prevention and control; radiological nursing; decontamination; radiation protection
Wilson et al. [35], 2022/USA	Frequency of deployed nursing skills required for sustaining a casualty for the first 72 h after injury in accordance with relevant nursing ICTL	Analytical cross-sectional study/analysis of dataset	Doctors/nurses (28,950)	Armed conflicts/pre-hospital and hospital	Blood gas interpretation; preparation for transfer to higher level of care; post-operative management; serious head injury management; administration of packed red blood cells; ventilator management; administration of fresh frozen plasma; arterial access; assistance with intubation; administration of platelets; administration of cryoprecipitate; chest tube placement; obtaining an ECG; central line maintenance; administration of whole blood or nasogastric tube; severe burn management; management of hemo-pneumothorax; management of ICP monitor; vasopressor infusion; cardiopulmonary resuscitation
Husna et al. [36], 2021/Indonesia	Perceptions, roles, and barriers to Islamic competencies in disaster response	Qualitative study/focus group	Nurses (24)	All disasters/hospital	Perception about disaster (religion/beliefs/values); communication skills (social interaction, nurse as patient’s advisor); nurses’ role in disaster response (the use of Islamic values in managing patients’ conditions/family engagement)
Ramirez-Miranda et al. [37], 2021/Peru	A professional profile by generic and specific competences for emergency and disaster nurses	Expert consensus/Delphi method	Disaster nurses	All disasters/not reported	Leadership capacity; emotional and social intelligence; initiative for decision-making; assertive communication and ability to resolve situations that compromise people’s lives; practice of values such as responsibility and solidarity with a respectful attitude towards the diversity, creeds and culture of the people concerned; diagnostic capacity and technical ability to provide specialized nursing care to the person in emergency and/or emergency situation; management of human and material resources for patient care
Rezaei et al. [38], 2020/Iran	Professional capabilities of nurses in providing care to the victims of the Kermanshah earthquake	Qualitative study/semi-structured interviews	Nurses (16)	Earthquakes/not reported	Clinical competence; personal competences; ethical competence; essential skills in caring for the injured
Ablah et al. [39], 2019/USA	An initial framework for guiding the public health workforce toward improved readiness for and proficient performance in emergencies	Expert consensus/Delphi method	Public health workers	All disasters/not reported	Model leadership; communication and management of information; protecting worker health and safety
Akbari et al. [40], 2018/Iran	Nurses’ perceptions of disaster competencies required by nurses based on their own experiences of working in a disaster	Qualitative study/semi-structured interviews	Nurses (35)	Earthquakes, flooding, and pandemics/not reported	Disaster scene coordination; human and other resource management
Prosdocimi & Witt [41], 2018/Brazil	PHC nurses’ competencies when responding to hydrological disasters in rural areas	Qualitative study/semi-structured interviews	Public healthcare nurses (20)	Floods/not reported	Leadership and management; teamwork; healthcare; community-oriented; communication; psychological care; health surveillance; educational
Al Thobaity et al. [42], 2017/Australia	The most common domains of the core competencies of nurses in disaster management	Scoping review	Nurses	All disasters/not reported	Communication; planning; decontamination and safety; ICS and ethics
Veenema et al. [43], 2017/USA	Essential competencies of nursing and hospital administrators’ leadership during disaster events	Qualitative study/focus group	Nurse executives (20)/hospital managers (33)	Urban disaster event/hospital	Problem solving and decision-making; leadership traits; personality traits and leadership styles; understanding organizational culture; general knowledge; human resources management communications; disaster management
Li et al. [44], 2016/China	Concepts and elements of disaster nursing, including disaster nursing skill requirements and architectural framework	Opinion study	Disaster nurses	All disasters/not reported	Emergency rescue competency; rational allocation and management of resources; psychological support and health education; medical device technology; interpersonal skills; critical thinking skills; good physical qualities
Marin & Witt [45], 2015/Brazil	Hospital nurses’ competencies in disaster situations	Qualitative study/focus group	Nurses (11)	All disasters/hospital	Management; healthcare; communication
Yan et al. [46], 2015/China	Skills, knowledge and attitudes required by RNs from across China who worked in the aftermath of three large earthquakes	Opinion study/survey	Nurses	Earthquakes/not reported	Clinical skills; psychological knowledge and skills; communication skills; self-protecting skills; immediate response skills; coordination skills; skills of infection control
Aliakbari et al. [47], 2014/Iran	Technical competencies required by nurses during disaster response	Qualitative study/semi-structured interviews	Nurses (30)	Earthquakes and all disasters/not reported	Basic and special nursing knowledge; skill to care for injured people
Bahrami et al. [48], 2014/Iran	Essential competences required to effectively respond to disaster situations	Qualitative study/semi-structured interview	Nurses (35)	All disasters/not reported	Management of the nursing response; ethical and legal competences; teamwork ability; specific personal ability; technical competence
Johnson et al. [49], 2013/USA	Knowledge and clinical skills that military healthcare providers might require in order to provide appropriate care for pediatric patients during civic assistance, humanitarian, and disaster relief efforts	Integrative review	Military healthcare providers	All disasters/not reported	Pediatric clinical skills and knowledge
Schultz et al. [50], 2012/USA	Disaster core competencies for acute care medical professionals	Expert consensus/instructional systems design	Emergency nurses/physicians/out-of-hospital EMS personnel	All disasters/not reported	Preparation and planning; detection and communication; incident management and support systems; clinical/public health assessment and intervention; contingency/continuity/and recovery; public health law and ethics
Yin et al. [51], 2011/China	Nurses’ basic care skills when part of first responder teams to the disaster site	Opinion study/survey	Nurses	Earthquakes/not reported	Mass casualty transportation; emergency management; hemostasis, bandaging, fixation, and manual handling; observation and monitoring; mass casualty triage; controlling specific infection; psychological crisis intervention; cardiopulmonary resuscitation; debridement and dressing; central venous catheter insertion; recording patient care

Legend. EMS: emergency medical services; RNs: registered nurses; PHC: primary healthcare; ICS: Incident Command System; ICP: intracranial pressure; ECG: electrocardiogram; ICTL: Individual Critical Task List; CBRNe: Chemical, Biological, Radiological, Nuclear and Explosive hazard.

## Data Availability

No new data were created or analyzed in this study.

## Supplementary Materials

The following supporting information can be downloaded at: https://www.mdpi.com/article/10.3390/nursrep16060206/s1. Files: S1, Preferred Reporting Items for Systematic reviews and Meta-Analyses extension for Scoping Reviews (PRISMA-ScR) checklist; S2, Search strategy. Tables: S1, Critical appraisal; S2, KSA competency matrix; S3, Variation across disaster type; S4, Variation across geographical area; S5, Variation across organizational setting; S6, Barriers; S7, Competency bases.

## Public Involvement Statement

No public involvement in any aspect of this research.

## Guidelines and Standards Statement

This manuscript was drafted against the Preferred Reporting Items for Systematic Reviews and Meta-Analysis extension for scoping reviews (PRISMA-ScR) guidelines [16].
