# Supplementary material for: Mapping Nursing Competencies Described for Disaster Response Within the Civil Defense Context: A Scoping Review
Source: Nurs Rep. 2026 Jun 18;16(6):206. doi: 10.3390/nursrep16060206 (PMC13304556; doi:10.3390/nursrep16060206)
Supplement: Supplementary file 1 [file nursrep-16-00206-s001.zip › nursrep-4321914-supplementary.pdf]

Supplementary Files

S1. Preferred Reporting Items for Systematic reviews and Meta-Analyses extension for Scoping Reviews (PRISMA-ScR) Checklist

SECTION	ITEM	PRISMA-ScR CHECKLIST ITEM	REPORTED ON PAGE #
**TITLE**			
Title	1	Identify the report as a scoping review.	✓
**ABSTRACT**			
Structured summary	2	Provide a structured summary that includes (as applicable): background, objectives, eligibility criteria, sources of evidence, charting methods, results, and conclusions that relate to the review questions and objectives.	✓
**INTRODUCTION**			
Rationale	3	Describe the rationale for the review in the context of what is already known. Explain why the review questions/objectives lend themselves to a scoping review approach.	3-4
Objectives	4	Provide an explicit statement of the questions and objectives being addressed with reference to their key elements (e.g., population or participants, concepts, and context) or other relevant key elements used to conceptualize the review questions and/or objectives.	4
**METHODS**			
Protocol and registration	5	Indicate whether a review protocol exists; state if and where it can be accessed (e.g., a Web address); and if available, provide registration information, including the registration number.	5
Eligibility criteria	6	Specify characteristics of the sources of evidence used as eligibility criteria (e.g., years considered, language, and publication status), and provide a rationale.	5
Information sources*	7	Describe all information sources in the search (e.g., databases with dates of coverage and contact with authors to identify additional sources), as well as the date the most recent search was executed.	5 + ST1
Search	8	Present the full electronic search strategy for at least 1 database, including any limits used, such that it could be repeated.	ST1
Selection of sources of evidence†	9	State the process for selecting sources of evidence (i.e., screening and eligibility) included in the scoping review.	6
Data charting process‡	10	Describe the methods of charting data from the included sources of evidence (e.g., calibrated forms or forms that have been tested by the team before their use, and whether data charting was done independently or	7

SECTION	ITEM	PRISMA-ScR CHECKLIST ITEM	REPORTED ON PAGE #
		in duplicate) and any processes for obtaining and confirming data from investigators.	
Data items	11	List and define all variables for which data were sought and any assumptions and simplifications made.	6-7
Critical appraisal of individual sources of evidence§	12	If done, provide a rationale for conducting a critical appraisal of included sources of evidence; describe the methods used and how this information was used in any data synthesis (if appropriate).	6
Synthesis of results	13	Describe the methods of handling and summarizing the data that were charted.	6-7
**RESULTS**			
Selection of sources of evidence	14	Give numbers of sources of evidence screened, assessed for eligibility, and included in the review, with reasons for exclusions at each stage, ideally using a flow diagram.	7 + Table 1
Characteristics of sources of evidence	15	For each source of evidence, present characteristics for which data were charted and provide the citations.	7-9 (Table 1)
Critical appraisal within sources of evidence	16	If done, present data on critical appraisal of included sources of evidence (see item 12).	ST2
Results of individual sources of evidence	17	For each included source of evidence, present the relevant data that were charted that relate to the review questions and objectives.	10-13 (Table 2)
Synthesis of results	18	Summarize and/or present the charting results as they relate to the review questions and objectives.	14-16
**DISCUSSION**			
Summary of evidence	19	Summarize the main results (including an overview of concepts, themes, and types of evidence available), link to the review questions and objectives, and consider the relevance to key groups.	17-19
Limitations	20	Discuss the limitations of the scoping review process.	19
Conclusions	21	Provide a general interpretation of the results with respect to the review questions and objectives, as well as potential implications and/or next steps.	19-20
**FUNDING**			
Funding	22	Describe sources of funding for the included sources of evidence, as well as sources of funding for the scoping review. Describe the role of the funders of the scoping review.	Title Page - 1

S2. Search strategy

Database	Limits	Total	Query
PubMed 27/07/2025	/	2,792	("nurses"[MeSH Terms] OR "nursing"[MeSH Terms] OR "nurs*"[All Fields] OR "register* nurs*"[All Fields] OR "nursing staff"[MeSH Terms] OR "nurs* staff"[All Fields] OR "nursing personnel"[All Fields]) AND ("competenc*"[All Fields] OR "skill*"[All Fields] OR "clinical competence"[MeSH Terms] OR "clinical competenc*"[All Fields] OR "professional competence"[MeSH Terms] OR "professional competenc*"[All Fields] OR "cultural competency"[MeSH Terms] OR "social skills"[MeSH Terms] OR "clinical competence"[MeSH Terms] OR "clinical skill*"[All Fields]) AND ("civil defense"[MeSH Terms] OR "civil defense"[All Fields] OR "civil defense*"[All Fields] OR "civil protection"[All Fields] OR "civil protection*"[All Fields] OR "disaster medicine"[MeSH Terms] OR "disaster nursing"[MeSH Terms] OR "disaster nurs*"[All Fields] OR "disaster response"[All Fields] OR "disasters"[MeSH Terms] OR "rescue work"[MeSH Terms] OR "relief work"[MeSH Terms] OR "humanitarian assistance"[All Fields] OR "natural disasters"[MeSH Terms] OR "natural phenomen*"[All Fields] OR "environmental disaster*"[All Fields] OR "avalanches"[MeSH Terms] OR "avalanches"[All Fields] OR "cyclonic storms"[MeSH Terms] OR "cyclonic storms"[All Fields] OR "droughts"[MeSH Terms] OR "droughts"[All Fields] OR "earthquakes"[MeSH Terms] OR "earthquakes"[All Fields] OR "floods"[MeSH Terms] OR "floods"[All Fields] OR "landslides"[MeSH Terms] OR "landslides"[All Fields] OR "tidal waves"[MeSH Terms] OR "tidal waves"[All Fields] OR "tornadoes"[MeSH Terms] OR "tornadoes"[All Fields] OR "tsunamis"[MeSH Terms] OR "tsunamis"[All Fields] OR "wildfires"[MeSH Terms] OR "wildfires"[All Fields] OR "man made phenomen*"[All Fields] OR "manmade disasters"[All Fields] OR "terrorism"[MeSH Terms] OR "terrorism"[All Fields] OR "terroris*"[All Fields] OR "mass casualty incidents"[MeSH Terms] OR "mass shooting events"[MeSH Terms] OR "nuclear accidents"[All Fields] OR "bioterrorism"[MeSH Terms] OR "bioterrorism"[All Fields] OR "bioterroris*"[All Fields] OR "chemical accidents"[All Fields] OR "explosions"[MeSH Terms] OR "explosions"[All Fields] OR "fires"[MeSH Terms] OR "fires"[All Fields] OR "plane crashes"[All Fields] OR "train accidents"[All Fields] OR "armed conflicts"[MeSH Terms] OR "wars"[All Fields] OR "epidemics"[MeSH Terms] OR "epidemics"[All Fields] OR "infectious disease outbreaks"[All Fields] OR "humanitarian crisis"[All Fields])
Cochrane Library 27/07/2025	/	161	([mh nurses] OR [mh nursing] OR nurs* OR (register* NEXT nurs*) OR [mh "nursing staff"] OR (nurs* NEXT "staff") OR "nursing personnel")) AND ((competenc* OR skill* OR [mh "clinical competence"] OR ("clinical" NEXT competenc*) OR [mh "professional competence"] OR ("professional" NEXT competenc*) OR [mh "cultural competency"] OR [mh "social skills"] OR [mh "clinical competence"] OR ("clinical" NEXT skill*)) AND ([mh "civil defense"] OR "civil defense" OR ("civil" NEXT defense*) OR "civil protection" OR ("civil" NEXT protection*) OR [mh "disaster medicine"] OR [mh "disaster nursing"] OR ("disaster" NEXT nurs*) OR "disaster response" OR [mh disasters] OR [mh "rescue work"] OR [mh "relief work"] OR "humanitarian assistance" OR [mh "natural disasters"] OR ("natural" NEXT phenomen*) OR ("environmental" NEXT disaster*) OR [mh avalanches] OR avalanches OR [mh "cyclonic storms"] OR "cyclonic storms" OR [mh droughts] OR droughts OR [mh earthquakes] OR earthquakes OR [mh floods] OR floods OR [mh landslides] OR landslides OR [mh "tidal waves"] OR "tidal waves" OR [mh tornadoes] OR tornadoes OR [mh tsunamis] OR tsunamis OR [mh wildfires] OR wildfires OR ("man made" NEXT phenomen*) OR "man made disasters" OR [mh terrorism] OR terrorism OR terroris* OR [mh "mass casualty incidents"] OR [mh "mass shooting events"] OR "nuclear accidents" OR [mh bioterrorism] OR bioterrorism OR bioterroris* OR "chemical accidents" OR [mh explosions] OR explosions OR [mh fires] OR fires OR "plane crashes" OR "train accidents" OR [mh "armed conflicts"] OR wars OR [mh epidemics] OR epidemics OR "infectious disease outbreaks" OR "humanitarian crisis")
Embase 07/08/2025	/	1,106	('nurse'/exp OR 'nursing'/exp OR nurs*:ti,ab OR 'registered nurs*':ti,ab OR 'nursing staff'/exp OR 'nursing staff':ti,ab OR 'nursing personnel':ti,ab) AND (competenc*:ti,ab OR skill*:ti,ab OR 'clinical competence'/exp OR 'clinical competenc*':ti,ab OR 'professional competence'/exp OR 'professional competenc*':ti,ab OR 'cultural competency'/exp OR 'social skills'/exp OR 'clinical skill*':ti,ab) AND ('civil defense'/exp OR 'civil defense':ti,ab OR 'civil protection':ti,ab OR 'disaster medicine'/exp OR 'disaster nursing'/exp OR 'disaster nurs*':ti,ab OR 'disaster response':ti,ab OR 'disaster'/exp OR 'rescue work'/exp OR 'relief work'/exp OR 'humanitarian assistance':ti,ab OR 'natural disaster'/exp OR 'natural phenomen*':ti,ab OR 'environmental disaster*':ti,ab OR 'avalanche'/exp OR avalanches:ti,ab OR 'cyclone'/exp OR 'cyclonic storms':ti,ab OR 'drought'/exp OR droughts:ti,ab OR 'earthquake'/exp OR earthquakes:ti,ab OR 'flood'/exp OR floods:ti,ab OR 'landslide'/exp OR landslides:ti,ab OR 'tidal wave'/exp OR 'tidal waves':ti,ab OR 'tornado'/exp OR tornadoes:ti,ab OR 'tsunami'/exp OR tsunamis:ti,ab OR 'wildfire'/exp OR wildfires:ti,ab OR 'man-made disaster':ti,ab OR 'man-made phenomen*':ti,ab OR

			'terrorism'/exp OR terroris*:ti,ab OR 'mass casualty incident'/exp OR 'mass shooting event'/exp OR 'nuclear accident':ti,ab OR 'bioterrorism'/exp OR bioterroris*:ti,ab OR 'chemical accident':ti,ab OR 'explosion'/exp OR explosions:ti,ab OR 'fire'/exp OR fires:ti,ab OR 'plane crash':ti,ab OR 'train accident':ti,ab OR 'armed conflict'/exp OR wars:ti,ab OR 'epidemic'/exp OR epidemics:ti,ab OR 'infectious disease outbreak':ti,ab OR 'humanitarian crisis':ti,ab)
Scopus 07/08/2025	/	1,748	(TITLE-ABS-KEY (nurs*) OR TITLE-ABS-KEY ("registered nurs*") OR TITLE-ABS-KEY ("nursing staff") OR TITLE-ABS-KEY ("nursing personnel")) AND (TITLE-ABS-KEY (competenc*) OR TITLE-ABS-KEY (skill*) OR TITLE-ABS-KEY ("clinical competenc*") OR TITLE-ABS-KEY ("professional competenc*") OR TITLE-ABS-KEY ("cultural competenc*") OR TITLE-ABS-KEY ("social skills") OR TITLE-ABS-KEY ("clinical skill*")) AND (TITLE-ABS-KEY ("civil defense") OR TITLE-ABS-KEY ("civil protection") OR TITLE-ABS-KEY ("disaster nurs*") OR TITLE-ABS-KEY ("disaster response") OR TITLE-ABS-KEY ("humanitarian assistance") OR TITLE-ABS-KEY ("natural phenomen*") OR TITLE-ABS-KEY ("environmental disaster*") OR TITLE-ABS-KEY (avalanches) OR TITLE-ABS-KEY ("cyclonic storms") OR TITLE-ABS-KEY (droughts) OR TITLE-ABS-KEY (earthquakes) OR TITLE-ABS-KEY (floods) OR TITLE-ABS-KEY (landslides) OR TITLE-ABS-KEY ("tidal waves") OR TITLE-ABS-KEY (tornadoes) OR TITLE-ABS-KEY (tsunamis) OR TITLE-ABS-KEY (wildfires) OR TITLE-ABS-KEY ("man-made phenomen*") OR TITLE-ABS-KEY ("man-made disasters") OR TITLE-ABS-KEY (terrorism) OR TITLE-ABS-KEY (terroris*) OR TITLE-ABS-KEY ("mass casualty incidents") OR TITLE-ABS-KEY ("mass shooting events") OR TITLE-ABS-KEY ("nuclear accidents") OR TITLE-ABS-KEY (bioterroris*) OR TITLE-ABS-KEY ("chemical accidents") OR TITLE-ABS-KEY (explosions) OR TITLE-ABS-KEY (fires) OR TITLE-ABS-KEY ("plane crashes") OR TITLE-ABS-KEY ("train accidents") OR TITLE-ABS-KEY (wars) OR TITLE-ABS-KEY (epidemics) OR TITLE-ABS-KEY ("infectious disease outbreaks") OR TITLE-ABS-KEY ("humanitarian crisis"))
CINAHL 27/07/2025	/	1,454	((MH nurses+) OR (MH nursing+) OR nurs* OR "register* nurs*" OR (MH "nursing staff+") OR "nurs* staff" OR "nursing personnel") AND (competenc* OR skill* OR (MH "clinical competence+") OR "clinical competenc*" OR (MH "professional competence+") OR "professional competenc*" OR (MH "cultural competency+") OR (MH "social skills+") OR (MH "clinical competence+") OR "clinical skill*") AND ((MH "civil defense+") OR "civil defense" OR "civil defense*" OR "civil protection" OR "civil protection*" OR (MH "disaster medicine+") OR (MH "disaster nursing+") OR "disaster nurs*" OR "disaster response" OR (MH disasters+) OR (MH "rescue work+") OR (MH "relief work+") OR "humanitarian assistance" OR (MH "natural disasters+") OR "natural phenomen*" OR "environmental disaster*" OR (MH avalanches+) OR avalanches OR (MH "cyclonic storms+") OR "cyclonic storms" OR (MH droughts+) OR droughts OR (MH earthquakes+) OR earthquakes OR (MH floods+) OR floods OR (MH landslides+) OR landslides OR (MH "tidal waves+") OR "tidal waves" OR (MH tornadoes+) OR tornadoes OR (MH tsunamis+) OR tsunamis OR (MH wildfires+) OR wildfires OR "man made phenomen*" OR "man made disasters" OR (MH terrorism+) OR terrorism OR terroris* OR (MH "mass casualty incidents+") OR (MH "mass shooting events+") OR "nuclear accidents" OR (MH bioterrorism+) OR bioterrorism OR bioterroris* OR "chemical accidents" OR (MH explosions+) OR explosions OR (MH fires+) OR fires OR "plane crashes" OR "train accidents" OR (MH "armed conflicts+") OR wars OR (MH epidemics+) OR epidemics OR "infectious disease outbreaks" OR "humanitarian crisis")

Supplementary Tables

S1. Critical Appraisal

STUDY DESIGN	APPRAISAL
Systematic Reviews and Research Synteses	79%
Analytical Cross-sectional Studies	75%
Qualitative Research	82%
Text and Opinion Studies	87%

S2. KSA Competency Matrix.

Study	CLIN	TRI	IPC	PSY	COM	LEAD	CULT	ETH	CBRNe	SURV	DOC	ADV
Rizek 2025	K S	S		K	S	S		A				
Wu et al. 2025	K		K S A	A	S				K S	K		
Mani et al. 2024	S	S		A	S	S	A	K				
Pierre et al. 2024	K S	S	K	S A	S	K						
Salik et al. 2024				A	S			A				
Santos et al. 2024	K S	K S	K	S A	K S	K S	K A	K	K	K	K	
Mani et al. 2023	S	S		A	S	S						
Silva et al. 2023	K							K			S	S
Lin et al. 2022					K S		K S A					
Su et al. 2022	S	S	S									
Wilson et al. 2022	S					S					S	S
Husna et al. 2021				A	S		A	A				
Ramirez-Miranda et al. 2021	K	S	K			S			K	S	S	
Rezaei et al. 2020	S	S		A	S	S		A				
Ablah et al. 2019					K	K S				K		
Akbari et al. 2018				S A	S	S						
Prosdocimi & Witt 2018					K S	S				K		

Al Thobaity et al. 2017	K S	S							K S			
Veenema et al. 2017	K				A	S	K S A					
Li et al. 2016	K	S			S	S						
Marin & Witt 2015					S	S		A				
Yan et al. 2015	K	S		A								
Aliakbari et al. 2014	S					S		K A	S			
Bahrami et al. 2014				A				A				
Johnson et al. 2013	K S		S						K S			
Schultz et al. 2012	K	S	K	K	K	K	K	K	K S	K		
Yin et al. 2011	S	S										
FREQUENCY	19	13	7	13	17	16	6	11	7	6	4	2
PERCENT (%)	70%	48%	26%	48%	63%	60%	22%	41%	26%	22%	15%	7%

Legend. *K=Knowledge; S=Skill; A=Attitude; CLIN=Clinical; TRI=Triage; IPC=Infection Prevention and Control; PSY=Psychological; COM=Communication; LEAD=Leadership; CULT=Cultural; ETH=Ethical; CBRNe=Chemical, Biological, Radiological, Nuclear and Explosive hazard; SURV=Surveillance; DOC=Documentation; ADV=Advanced* ***competencies.***

S3. Variation across Disaster Type

Disaster Type	Most Reported	Less Reported	Distinctive Patterns
Earthquakes	CLIN ǀ TRI ǀ PSY	DOC ǀ LEAD	Focus on technical survival skills (S) and physical trauma
Floods	IPC ǀ SURV ǀ COM	ADV ǀ TRI	Focus on community monitoring and waterborne disease prevention
Armed Conflicts	CLIN ǀ TRI ǀ ETH ǀ ADV	CULT ǀ DOC	Focus on moral resilience (A) and trauma care in hostile areas
Biosafety	IPC ǀDOC	TRI ǀ LEAD	Focus on strict safety compliance (K) and containment
All-Hazard	COM ǀ LEAD ǀ ETH ǀ PSY	CLIN ǀ SURV	Focus on portable core skills applicable across any scenario

Legend. *CLIN=Clinical; TRI=Triage; IPC=Infection Prevention and Control; PSY=Psychological; COM=Communication; LEAD=Leadership; CULT=Cultural; ETH=Ethical; CBRNe=Chemical, Biological, Radiological, Nuclear and Explosive hazard; SURV=Surveillance; DOC=Documentation; ADV=Advanced competencies; K=Knowledge; S=Skill; A=Attitude*

S4. Variation across Geographical Area

**Geographical Area**	**Most Reported**	**Less Reported**	**Contextual Characteristics**
Asia	CLIN ǀ PSY ǀ CULT	LEAD ǀ ADV	High exposure to natural disasters (Ring of Fire); strong influence of spiritual values and community resilience
Middle East	TRI ǀ CLIN ǀ ETH	CULT ǀ SURV	Persistent political instability and armed conflict; requirement to manage blast injuries and massive refugee influxes
South America	LEAD ǀ DOC ǀ CLIN	TRI ǀ IPC	Focus on territorial coordination, legal aspects of disaster response, and forensic nursing in the post-disaster phase

West (USA/EU)	LEAD ǀ COM ǀ IPC	PSY ǀ CULT	Highly structured health systems; emphasis on standardization, All-Hazard protocols and Incident Command Systems (ICS)

Legend. *CLIN=Clinical; TRI=Triage; IPC=Infection Prevention and Control; PSY=Psychological; COM=Communication; LEAD=Leadership; CULT=Cultural; ETH=Ethical; CBRNe=Chemical, Biological, Radiological, Nuclear and Explosive hazard; SURV=Surveillance; DOC=Documentation; ADV=Advanced competencies.*

S5. Variation across Organizational Setting

Setting	Most Reported	Less Reported	Role Characteristics
Hospital (ED/ICU)	CLIN ǀ IPC ǀ LEAD	SURV ǀ CULT	Managing surge capacity, decontamination, clinical stability of critically ill patients
Pre-Hospital / Field	TRI ǀ COM ǀ CLIN	DOC ǀ ETH	Maximum decision-making autonomy; rapid rescue, field triage, logistical improvisation
Community Care	CULT ǀ PSY ǀ SURV	ADV ǀ TRI	Long-term support role; infectious disease prevention and psychological management of vulnerable populations
Military / Austere	ADV ǀ CLIN ǀ TRI	CULT ǀ LEAD	Specialized in Prolonged Casualty Care; managing injuries for extended periods without logistics or immediate evacuation

Legend: *CLIN=Clinical; TRI=Triage; IPC=Infection Prevention and Control; PSY=Psychological; COM=Communication; LEAD=Leadership; CULT=Cultural; ETH=Ethical; CBRNe=Chemical, Biological, Radiological, Nuclear and Explosive hazard; SURV=Surveillance; DOC=Documentation; ADV=Advanced competencies*

S6. Barriers

Study	Individual barriers		Organizational barriers		Systemic/Other barriers	
	Psychological Stress & Moral Distress	Lack of Prior Training/ Experience	Resource & Equipment Scarcity	Role Ambiguity & Lack of Protocols	Inconsistent Educational Frameworks	Cultural & Language Barriers
Rizek 2025	X	X		X	X	
Wu et al. 2025		X	X			
Mani et al. 2024	X					X
Pierre et al. 2024	X	X				
Salik et al. 2024					X	
Santos et al. 2024	X	X	X			
Mani et al. 2023			X			
Silva et al. 2023					X	
Lin et al. 2022		X			X	
Su et al. 2022		X	X			
Wilson et al. 2022						X
Husna et al. 2021	X		X			X
Ramirez-Miranda et al. 2021					X	
Rezaei et al. 2020	X					
Ablah et al. 2019			X			
Akbari et al. 2018				X	X	

Prosdocimi & Witt 2018	X			X		
Al Thobaity et al. 2017		X			X	
Veenema et al. 2017		X			X	
Li et al. 2016		X			X	
Marin & Witt 2015		X		X		
Yan et al. 2015		X				
Aliakbari et al. 2014						X
Bahrami et al. 2014				X	X	
Johnson et al. 2013					X	
Schultz et al. 2012	X			X		
Yin et al. 2011		X				
FREQUENCY	8	12	6	6	11	4
PERCENT (%)	30%	44%	22%	22%	41%	15%

S7. Competency Bases

Study	Primary Basis	Specific Framework or Source Referenced
Rizek 2025	FRA	ENA (Emergency Nurses Association) & NHDP-BC Standards
Wu et al. 2025	EXP	Delphi consensus on Biosafety Incident response
Mani et al. 2024	EMP	Scoping review of evidence-based care during earthquakes
Pierre et al. 2024	EMP I GUI	Phenomenological evidence aligned with ICN 2.0
Salik et al. 2024	GUI	ICN Core Competencies in Disaster Nursing Version 2.0
Santos et al. 2024	EMP	Empirical field surveys in conflict-affected zones
Mani et al. 2023	EMP	Field-based evidence from Yemen/war-zone nursing
Silva et al. 2023	FRA	JBI Methodology & Forensic Nursing professional standards
Lin et al. 2022	EMP	Systematic scoping review of global nursing skills
Su et al. 2022	EMP I GUI	US Army Joint Trauma System & Military Medical Evidence
Wilson et al. 2022	EMP	Empirical review of Asian psychological interventions
Husna et al. 2021	GUI	ICN Framework adapted for local Indonesian context
Ramirez-Miranda et al. 2021	FRA I POL	Peruvian National Competency Profile (Law/Policy)
Rezaei et al. 2020	EMP	Qualitative evidence from Iranian earthquake relief
Ablah et al. 2019	EMP	Empirical research on Primary Health Care (PHC) settings
Akbari et al. 2018	EMP I EXP	Concept mapping and focus groups with nurse leaders

Prosdocimi & Witt 2018	EMP	Qualitative field evidence from experienced responders
Al Thobaity et al. 2017	GUI	ICN Framework of Disaster Nursing Competencies (V 1.0)
Veenema et al. 2017	POL ∣ GUI	Chinese Health Ministry research and ICN standards
Li et al. 2016	EMP ∣ FRA	Post-earthquake educational gap analysis (China)
Marin & Witt 2015	POL ∣ GUI	Brazilian National Policy (Portaria 1600/2011) & ICN
Yan et al. 2015	EMP	Empirical analysis of ethical challenges in the field
Aliakbari et al. 2014	POL ∣ GUI	WHO and Sphere Project humanitarian standards
Bahrami et al. 2014	FRA ∣ POL	CDC Public Health Preparedness (PHEP) Capabilities
Johnson et al. 2013	EXP ∣ EMP	Delphi study (All-Hazard focus) for national standards
Schultz et al. 2012	POL ∣ EMP	Iranian national evaluation of disaster response
Yin et al. 2011	EMP	Direct field practice survey (Wenchuan earthquake)

Legend: FRA: Accreditation Frameworks; GUI: Guidelines; POL: Policies; EMP: Empirical Evidence; EXP: Expert Consensus.
